# Pomegranate Juice Extract Decreases Cisplatin Toxicity on Peripheral Blood Mononuclear Cells

**DOI:** 10.3390/medicines7100066

**Published:** 2020-10-15

**Authors:** Mohamad Nasser, Ziad Damaj, Akram Hijazi, Othmane Merah, Batoul Al-Khatib, Nadine Hijazi, Christine Trabolsi, Raghida Damaj, Mouhamad Nasser

**Affiliations:** 1 Doctoral School of Science and Technology, Research Platform for Environmental Science (PRASE), Lebanese University, Beirut P.O. Box 5, Lebanon; mohamednasser@hotmail.fr (M.N.); damajz@hotmail.fr (Z.D.); batool.khatib93@hotmail.com (B.A.-K.); nadinehijazi96@hotmail.com (N.H.); r.damaj@ul.edu.lb (R.D.); 2 Rammal Hassan Rammal Research Laboratory, Physiotoxicity (PhyTox), Faculty of Sciences, Lebanese University, Nabatieh 1700, Lebanon; 3 Laboratoire de Chimie Agroindustrielle, LCA, Université de Toulouse, INRA, 31030 Toulouse, France; 4 Département Génie Biologique, IUT A, Université Paul Sabatier, 32000 Auch, France; 5 Faculty of Medicine, Lebanese University, Beirut P.O. Box 5, Lebanon; christinetrabolsi9@gmail.com; 6 Department of Respiratory Medicine, National Coordinating Reference Centre for Rare Pulmonary Diseases, Louis Pradel Hospital, University Hospital of Lyon, 69677 Lyon, France; mohammad-nasser@hotmail.com; 7 Claude Bernard University Lyon 1, UMR754, IVPC, 69008 Lyon, France

**Keywords:** lung cancer, *Punica granatum*, cisplatin, A549 cells, PBMC

## Abstract

**Background:** Lung cancer is one of the most prevalent cancers worldwide. Chemotherapy regimens, targeted against lung cancer, are considered an effective treatment; albeit with multiple fatal side effects. An alternative strategy, nowadays, is using natural products. Medicinal plants have been used, in combination with chemotherapy, to ameliorate side effects. This study aims to investigate the antitumor effect of pomegranate juice (*Punica granatum*) on human lung adenocarcinoma basal epithelial cells (A549), to check the effect, when combined with low dose cisplatin (CDDP), at different doses. We also have evaluated the potential protective effect of pomegranate on normal peripheral blood mononuclear cells (PBMC). **Methods:** Phytochemical screening of the extract was done using standard classical tests. Total phenolic and sugar contents were determined using the Folin–Ciocalteu and anthrone reagents, respectively. The antioxidant activity of pomegranate was estimated by the 2,2-diphenyl-1-picrylhydrazyl (DPPH) method. The viability of A549 cells and PBMC was evaluated using the neutral red assay. **Results:** Our results demonstrated that *Punica granatum* or pomegranate juice (with different concentrations: 150, 300, 600 µg/mL) contained high levels of flavonoids, alkaloids, tanins, lignins, terpenoids, and phenols. The DPPH method showed that pomegranate juice had a strong antioxidant scavenging activity. Neutral red showed that combining pomegranate juice with low dose CDDP (8 µg/mL) decreased the cell viability of A549 cells, by 64%, compared to treatment with CDDP or pomegranate alone. When added to low dose CDDP, pomegranate increased the viability of normal PBMC cells by 46%. **Conclusions:** These results demonstrated that pomegranate could potentiate the anticancer effect of low dose CDDP on human lung adenocarcinoma cells (A549 cells) and could as well decrease its toxicity on PBMC.

## 1. Introduction

Lung cancer is the main leading cause of cancer-related death in men, and the second in women [1]. Patients with lung cancer have a five-year survival rate of 15.6% in the United States [1]. Lung cancer is thought to arise from the respiratory epithelial cells, and it is widely classified into two main types: small cell lung and non-small cell lung cancer, accounting for 15% and 85% of cases, respectively. Non-small cell lung cancer is further subdivided into: adenocarcinoma (38.5% of all lung cancer cases), squamous cell carcinoma (20%), and large cell carcinoma (2.9%) [1].

Surgery can be curative in early stages; however, around half of patients are diagnosed in advanced stage, when lung resection cannot be entertained [2]. In such cases, chemotherapy and radiation therapy are used to increase relapse-free survival, or as a part of compassionate treatment in patients with compression related manifestations [3].

An appreciable proportion of patients necessitate chemotherapy following surgery, known as ‘adjuvant chemotherapy’, to decrease recurrence rate. This is particularly true for patients with stage II and IIIA disease [2].

The chemotherapy treatment plan often consists of a combination of drugs. Most commonly used drugs are cisplatin (CDDP), carboplatin, docetaxel, gemcitabine, paclitaxel, vinorelbine, and pemetrexed [3]. CDDP or cis-diamminedichloroplatinum (II) is a well-known platinum-based chemotherapeutic agent, used for the treatment of different neoplasms as lung, bladder, head and neck, and ovarian malignancies. CDDP induces apoptosis in tumor cells through the inhibition of DNA synthesis and repair mechanisms. Nevertheless, CDDP has many side effects such as: severe kidney problems, allergic reactions, immunity disease, gastrointestinal disorders, hemorrhage, hearing loss, and hematological toxic effects, such as anaemia, leukopenia, neutropenia, and thrombocytopenia [4,5,6].

Some medicinal plants, owing to their anticancer properties, have been used for treatment of distinct cancers like lung, skin, and bladder cancers [7]. Lebanon is a country rich in medicinal plants; one such plant is pomegranate [8].

*Punica granatum* or pomegranate is an ancient edible fruit that has been used for centuries as a medicine. Recent studies demonstrated antioxidant, antimicrobial, anticarcinogenic, anti-inflammatory, and antiviral properties of this plant. These properties are due to the presence of phytochemicals, including phenolics (e.g., flavonoids, tannins), terpenoids, and alkaloids [9]. Polyphenols, such as flavonoids, are ubiquitously present in fruits and vegetables. It has evinced the ability to downregulate the expression of various genes, proteins, and signaling cascades that are responsible for tumor growth and progression; making them potential therapeutic agents for cancer patients [10]. Pomegranate exerts anticancer and antioxidant activities, which are generally attributed to its high content of polyphenols, and its role in neutralizing free radicals. Pomegranate juice and pomegranate extracts inhibit the growth of prostate cancer cells in in vitro cultures, and also inhibit cell proliferation and induce apoptosis in human breast cancer cells, pancreatic cancer cells, and even in colon and hepatocellular carcinoma cell lines [11]. Moreover, pomegranate polyphenol has potent anticancer activity in breast, lung, and cervical cancer cells [12].

In order to decrease chemotherapy-related toxicity, cisplatin dose is reduced; a strategy that would mitigate its antineoplastic activity. In a previous study of ours, we demonstrated that combining pomegranate to low dose cisplatin on human lung adenocarcinoma (A549) cell lines, decreased their cell viability in comparison to those treated with chemotherapy drugs alone [13]. Yet, the protective effect of pomegranate on peripheral blood mononuclear cells (PBMC) has not been studied.

## 2. Materials and Methods

### 2.1. Preparation of Pomegranate Fruit Extract

Pomegranate fruit was taken from South Lebanon (Nmairiyeh village) which is at a height of 400 m from the surface of water. The fruit was peeled and squeezed then filtered to obtain the pomegranate juice. The juice was placed on ice at −80 °C for about 2 h. It was then kept in the lyophilizer for 3 days to remove water and convert them to powder. The powder was stored in a desiccator at room temperature.

### 2.2. Chemical Tests

#### 2.2.1. Phytochemical Screening Test

Pomegranate juice was centrifuged at 2500 rpm for 5 min at a temperature of 22 °C, and then the supernatant was filtered by filter paper using Buchner vacuum. This filtrate was used for the qualitative detection of primary and secondary metabolites according to Nasser et al. [14] (Table 1).

#### 2.2.2. Antioxidant Test (DPPH Radical Scavenging Method)

DPPH (2,2-diphenyl-1-picrylhydrazyl) from Sigma-Aldrich, is composed of stable free radical molecules which has a strong purple color that can be measured spectrophotometrically. In the presence of compounds capable of transferring an electron or donating hydrogen, DPPH becomes discolored, indicating therefore the antioxidant activity of the tested compound. In this study, radical scavenging activity was determined [15]. DPPH solution was prepared by dissolving 0.0012 g of DPPH powder in 50 mL of methanol. The extract was prepared by dissolving 10 mg of pomegranate powder in 0.5 mL distilled water and 0.5 mL of methanol. After that, 2 mL of DPPH solution was mixed with 50 µL of the extract solution and the mixed solution was put in dark for 30 min. The absorbance was then read at 515 nm on UV–vis spectrophotometer. The antioxidant activity was calculated according to the equation

% Antioxidant activity = 100 × (ABS control − ABS sample)/ABS control

The absorbance ABS control is the absorbance of DPPH + solvent; ABS sample is the absorbance of the DPPH + sample, where the control was prepared by taking 2 mL of the prepared DPPH solution (DPPH + methanol) without the extract.

#### 2.2.3. Carbohydrates Test

Anthrone (10H-Anthracen-9-one) from Sigma-Aldrich, is a tricyclic aromatic ketone, used for colorimetric determination of carbohydrates. In this method, carbohydrates are dehydrated with concentrated H_2_SO_4_ to form furfural which condenses with anthrone to form a green color complex, measured spectrophotometrically ultimately. First, 100 mg of pomegranate powder were macerated in 5.25 mL of 80% ethanol for 12 h. This solution was then centrifuged at 4000 rpm for 10 min. The supernatant was then diluted 1000 times to obtain solution A. Solution B was prepared by dissolving 1 g of anthrone in 500 mL concentrated sulfuric acid. Solution B (4 mL) was added to solution A (2 mL) and vortexed for 1 min and then put at 92 °C for 8 min. The mixture was placed in dark for 30 min and then the absorbance was measured at 585 nm. The blank tube was prepared similarly; yet, 2 mL of solution A were used with 2 mL of distilled water.

#### 2.2.4. Phenol Content Test

Folin–Ciocalteu reagent was a mixture of phosphomolbdate and phosphotungtate used for colorimetric determination of phenolic compounds by the method of Farhan et al. [16], with some modifications. This test was performed through the following steps. First, the sample was prepared by dissolving 25 mg of pomegranate powder in 10 mL distilled water. Distilled water (3.16 mL) and Folin–Ciocalteu reagent (200 µL) were added to 40 µL of the prepared sample and vortexed together. After 5 min, 600 µL of Na_2_CO_3_ solution, prepared by dissolving 20 g Na_2_CO_3_ in 100 mL distilled water, were added to the vortexed mixture and then vortexed for 1 min. They were heated in water bath at 40 °C for 30 min. The absorbance was measured at 765 nm after cooling the sample for 10 min. The blank tube was prepared by similar steps but instead of 40 µL of prepared sample, 40 µL distilled water was used.

#### 2.2.5. Dry Matter Test

Biomass is usually determined on a dry matter basis, which is the weight of plant material after the moisture within it, had been extracted. In this test, 1 g of pomegranate powder was weighted and put in oven at 100 °C for 24 h. The percentage of dry matter was determined using the equation

% Dry matter = (M_2_ − M_0_) × 100/(M_1_ − M_0_)

where M_0_ is the mass of empty beaker, M_1_ is the mass of beaker with extract, and M_2_ is the mass of beaker with extract after drying in oven.

### 2.3. Cell Culture and Treatment

#### 2.3.1. A549 Cells

The A549 cells, obtained from ATCC, Manassas, VA, USA, were grown in T75 cell culture flasks in a total volume of 10 mL of Dulbecco’s modified Eagle medium (DMEM) and supplemented with 10% fetal bovine serum, 1% penicillin/streptomycin and kept at 37 °C, 5% CO_2_ in humidified air in an incubator.

#### 2.3.2. Control Normal Cells

Peripheral blood mononuclear cells (PBMC), were isolated from human healthy volunteers blood and then underwent density gradient centrifugation. Briefly, blood is diluted by PBS in a 1:1 ratio. A 4 mL of diluted blood were added to 2 mL ficoll, and centrifuged at 400× *g* for 30 min without brake. Carefully then isolate the buffy coat layer containing PBMC. Washed PBMC by 5 mL PBS and centrifuged at 300× *g* for 15 min. Supernatant was removed and washed second time by 5mL, then centrifuged at 200× *g* for 15 min. The cells obtained were resuspended in RPMI medium supplemented with 10% fetal bovine serum, 1% penicillin/streptomycin, and seeded in 24-well plates and then incubated at 37 °C with 5% CO_2_ humidified air.

#### 2.3.3. Treatment of Cells

Cells’ treatment was done by lyophilized pomegranate dissolved in medium specific for each cell type by vortex and filtered by a filter of 0.22 µm diameter. Cells were seeded in a 24-well plate at a concentration of 100,000 cells/well then treated with different concentrations of pomegranate, CDDP (from Mylan-France), and combination of both.

The evaluation of antiproliferative activity was done by measuring cell viability of A549 cells and PBMC (control cells) after 24 h of treatment with increasing concentrations of CDDP (2, 4, 8, and 12 µg/mL) and with pomegranate juice extract (150, 300, and 600 µg/mL). Moreover, cells were treated with different combinations of pomegranate juice (150 and 300 µg/mL) and CDDP (2, 4, and 8 µg/mL).

### 2.4. Viability Tests

#### Neutral Red Uptake Assay

Cell viability was measured by performing neutral red assay. Briefly, after 24 h of treatment of A549 cells, medium was aspirated from wells and washed one time with phosphate buffered saline (PBS) then incubated for 3 h in a medium containing neutral red solution (40 µg/mL) from Sigma-Aldrich. Then the media were discarded and the cells were washed twice with PBS. After that, cells were incubated for few minutes in lysing solution (200 µL/well) formed of 1% acetic acid and 50% ethanol to extract the dye.

PBMC were spined and the medium was removed. Cells were incubated in each well in neutral red solution (40 µg/mL) for 3 h. After incubation, cells were spined down and the staining solution was removed then washed once with PBS and spined to remove washing solution. Then, lysing solution (200 µL/well) was used for A549 cells and incubated for few minutes.

The solutions of each well were then transferred to 96-well plate and read at 450 nm by microplate reader [17].

### 2.5. Statistical Analysis

Statistical analysis was performed using GraphPad Prism 5. Each value represents the mean ± the standard error of the mean (SEM). One-way ANOVA was used to assess cell viability, and differences between groups were analyzed by Bonferroni test. Groups that are significantly different from the control are indicated in the Figures as * *p* < 0.05, ** *p* < 0.01, and *** *p* < 0.001.

## 3. Results

### 3.1. Phytochemical Screening Test

Phytochemicals are bioactive molecules present in plants and involved in protecting human health. Qualitative tests of these compounds showed the presence of alkaloids, terpenoids, as well as phenols, including tannins, flavonoids and anthocyanins. Results of these tests are represented in Table 2.

### 3.2. Antioxidant Test (DPPH Radical Scavenging Method)

The antioxidant activity test showed that as the concentration of pomegranate juice increases the antioxidant activity increases too. This was observed by the decrease of violet color intensity as the concentration was increased from 10 to 20 mg/mL. The DPPH radical scavenging activity was 35.56 ± 0.89 TE µmol/g of extract. The optical density decreased as the concentration of pomegranate juice increased. In addition, the antioxidant activity of DPPH was more efficient with methanol than with ethanol. This antioxidant activity was 70% with methanol compared to 33% with ethanol knowing that methanol is more polar than ethanol and can extract more chemicals [18]. This was also observed by the transformation of violet color to yellow color as the concentration of DPPH was increased from 0.1 to 0.5 mg/mL.

### 3.3. Carbohydrate Test

Anthrone is widely used as a reagent in the quantitative determination of carbohydrates in plants. This test showed the presence of high amount of carbohydrates in pomegranate juice in which it was 69.29 ± 2.5% (Table 3). 

### 3.4. Phenol Content Test

The total phenol content of pomegranate juice extract was estimated using Folin–Ciocalteu assay. It was found to be 6.93 ± 0.09 mg GAE/g of pomegranate juice extract (Table 3).

### 3.5. Dry Matter Test

The most common way to determine is through the evaporation of water from the extract. The percentage of dry matter was 81.1463% (Table 3).

### 3.6. Cell Viability Results

#### 3.6.1. Neutral Red Assay

The optical density decreased after treatment as a function of concentration. Viable cells can take up neutral red via active transport and incorporate the dye into their lysosomes; whereas non−viable cells cannot take up this chromophore. The decrease in optical density for treated wells compared to control reflects the decrease in cell viability after treatment.

#### 3.6.2. Treatment of A549 Cells with CDDP in a Dose−Dependent Manner

**Figure 1:**
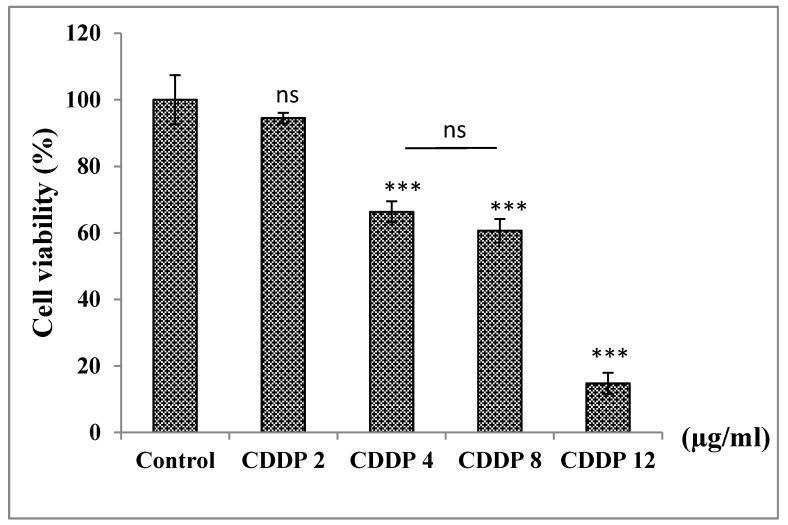
Effect of CDDP on cell viability of A549 cells after 24 h of treatment. ns: non significant *p* > 0.05, *** *p* < 0.001 in comparison to control. The number of samples is *n* = 6. (CDDP:cisplatin).

Treatment with CDDP for 24 h at increased concentrations (2, 4, 8, and 12 µg/mL) caused a decrease in cell viability of A549 cells compared to untreated cells (control). At concentration 2 µg/mL, cell viability decreased by 6% but there was no significant difference in comparison to the control. On the other hand, a significant decrease in cell viability compared to the control was seen for cells treated with concentrations 4, 8, and 12 µg/mL by 34%, 40%, and 85%, respectively (Figure 1).

#### 3.6.3. Treatment of A549 Cells with Pomegranate in a Dose−Dependent Manner

**Figure 2:**
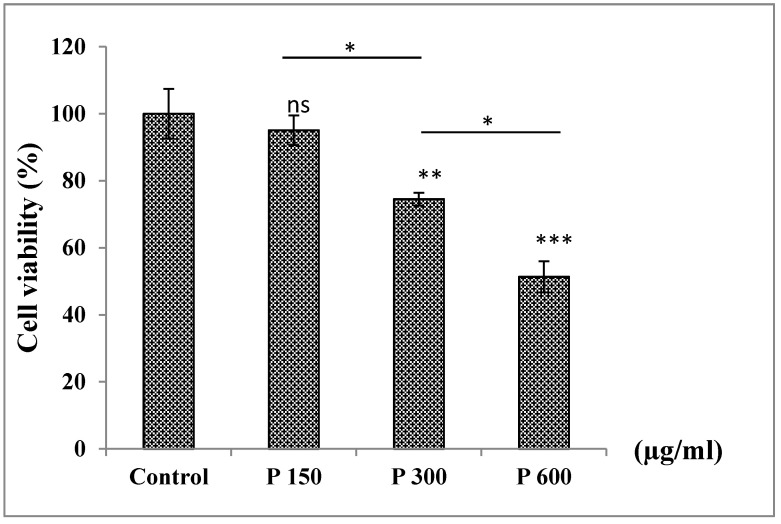
Effect of pomegranate on cell viability of A549 cells after 24 h of treatment. ns: non significant * *p* > 0.05, ** *p* < 0.01, *** *p* < 0.001 in comparison to the control. The number of samples is *n* = 6. (P: pomegranate).

Treatment of A549 cells with pomegranate juice extract at increased concentrations showed a decrease in cell viability. No significant difference was observed for concentration 150 µg/mL in which cell viability was decreased by 5% only, however, a significant decrease was seen with higher concentrations of 300 and 600 µg/mL (26% and 49%, respectively) (Figure 2).

#### 3.6.4. Effect of Combination of Pomegranate with CDDP on A549 Cell Viability

**Figure 3:**
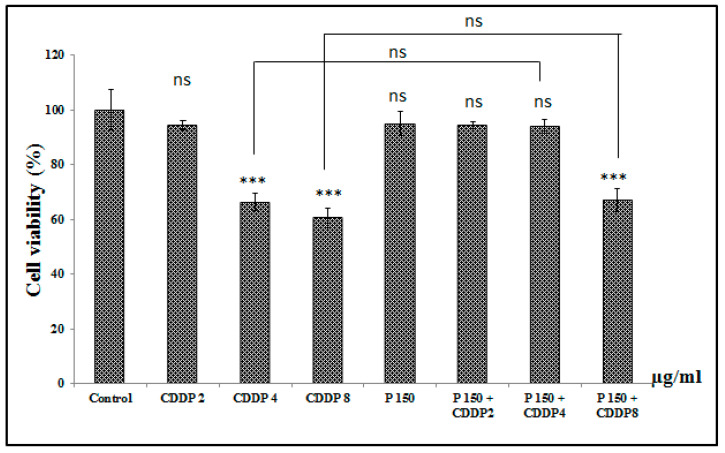
Effect of combining pomegranate (150 µg/mL) with CDDP on cell viability of A549 cells after 24 h of treatment. ns: non significant *p* > 0.05, *** *p* < 0.001 in comparison to control. The number of samples is *n* = 6 (CDDP: cisplatin, P: pomegranate juice).

Neutral red results showed decreased cell viability when pomegranate was combined with CDDP compared to control. Cells treated with 150 µg/mL pomegranate extract combined to 2, 4, and 8 µg/mL of CDDP showed no significant decrease in cell viability compared to CDDP alone (2, 4, and 8 µg/mL). However, at this concentration (150 µg/mL), pomegranate juice showed a proliferative effect. After combination of pomegranate (150 µg/mL) with 8 µg/mL of CDDP, there was a debate between pomegranate at this concentration and the antiproliferative effect of 8 µg/mL CDDP (Figure 3).

**Figure 4:**
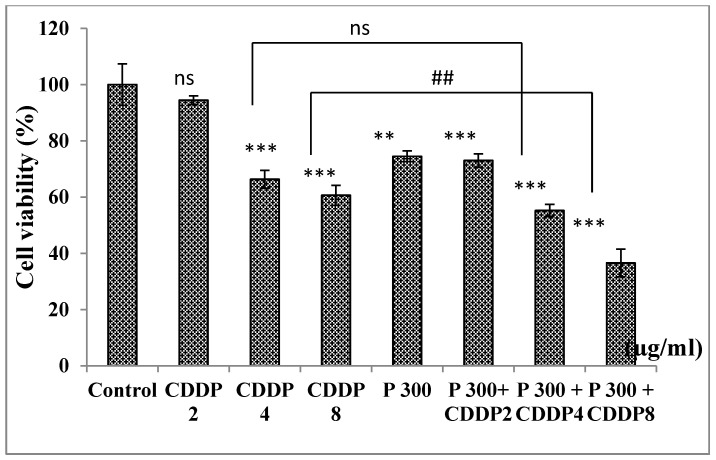
Effect of combining pomegranate (300 µg/mL) with CDDP on cell viability of A549 cells after 24 h of treatment. ns: non significant *p* > 0.05, ** *p* < 0.01, *** *p* < 0.001 in comparison to control, ## *p* < 0.01. The number of samples is *n* = 6. (CDDP: cisplatin, P: pomegranate juice).

On the other hand, treatment with pomegranate (300 µg/mL) combined with the three concentrations of CDDP (2, 4, and 8 µg/mL) showed a significant decrease in cell viability with a percentage decrease of 27%, 45%, and 64%, respectively. In addition, a significant difference was observed for the combination of pomegranate (300 µg/mL) with CDDP (8 µg/mL) in comparison to CDDP (8 µg/mL) alone. As well, there was a significant difference between pomegranate (300 µg/mL) alone and the combination with CDDP (8 µg/mL). Then, this concentration 300 µg/mL is the optimal concentration used in combination with CDDP 8 for the anti−cancer effect (Figure 4).

#### 3.6.5. Effect of Combination of Pomegranate and CDDP on Normal PBMC

**Figure 5:**
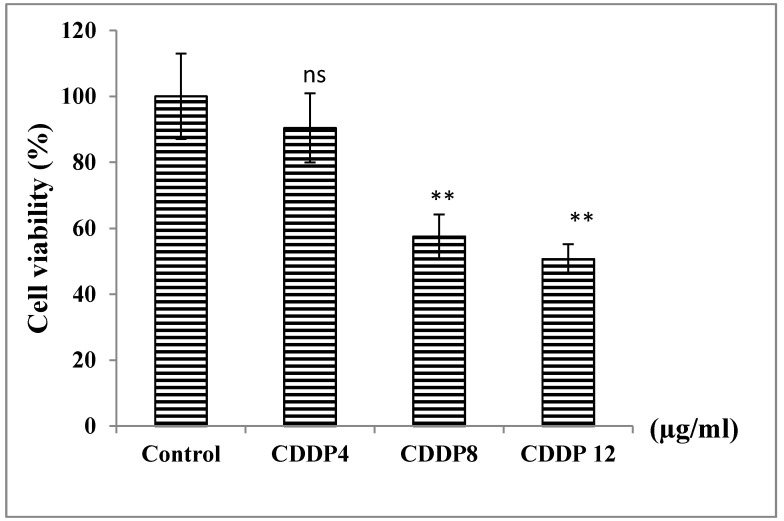
Effect of CDDP on cell viability of control normal PBMC cells after 24 h of treatment. ns: non significant *p* > 0.05, ** *p* < 0.01 by comparison to the control. The number of samples is *n* = 4 (CDDP: cisplatin).

Treatment with increased concentrations of CDDP for 24 h showed a significant decrease in cell viability of PBMC for concentrations 8 and 12 µg/mL with a percentage decrease of 43% and 50%, respectively (Figure 5).

**Figure 6:**
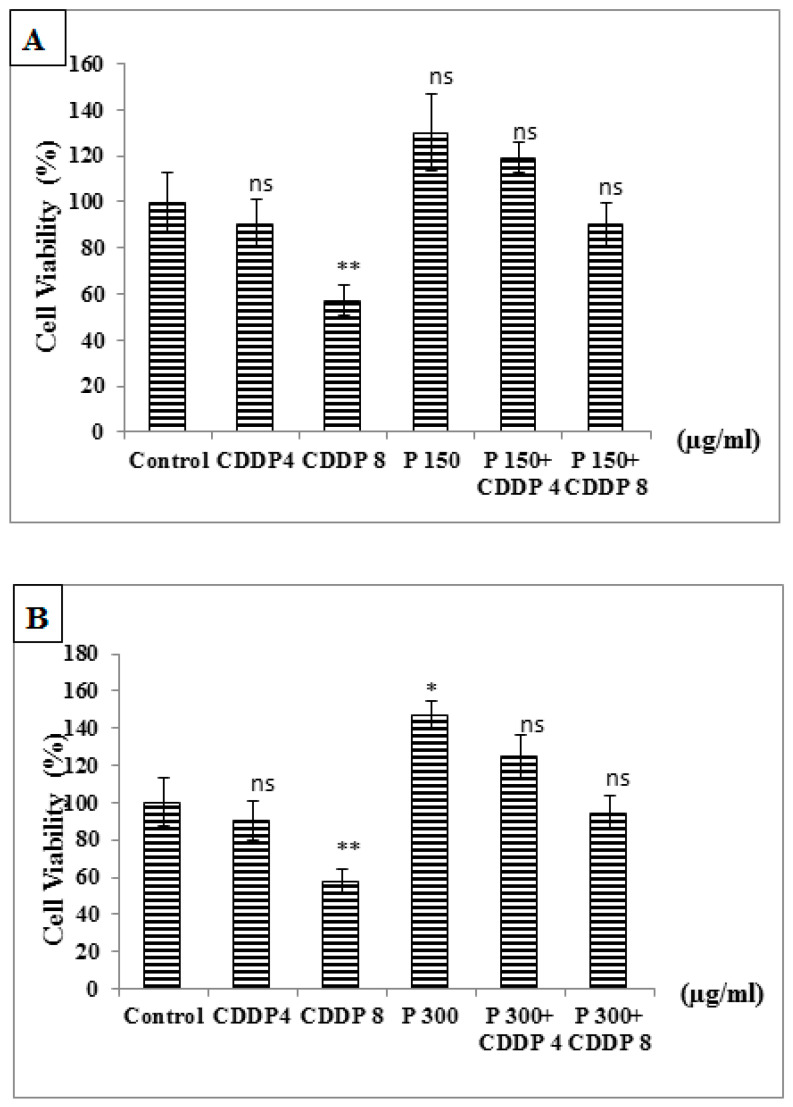
(**A**,**B**) Effect of combining pomegranate with CDDP on cell viability of control normal PBMC cells after 24 h of treatment. ns: non significant *p* > 0.05, * *p* < 0.05, ** *p* < 0.01 in comparison to control. The number of samples is *n* = 4. (CDDP: cisplatin, P: pomegranate juice).

However, treatment with pomegranate alone, or in combination with CDDP, had no significant effect on cell viability. Increased concentration of pomegranate (300 µg/mL) had a significant increase in PBMC cell viability by 46% compared to untreated cells (control) (Figure 6A,B).

## 4. Discussion

Lung cancer is the leading cause of cancer−related mortality around the world. About half of patients diagnosed with lung cancer die within one year of diagnosis. Several therapeutic modalities have been used recently to treat cancer patients such as surgery, chemotherapy, radiotherapy, and even targeted therapies. Among these, chemotherapy is one of the main treatments used for cancer patients; but this treatment causes many side effects that range from mild to severe, and may be even fatal [19].

Different studies on pomegranate have revealed its antineoplastic effect [20,21,22]. Patients treated with CDDP alone or in combination with other chemotherapeutic drugs may be exposed to serious adverse effects [4,23,24]. Hence, the goal of our study is to minimize the dose of CDDP and compensate it by combining pomegranate juice extract.

In this study, the combination of pomegranate juice extract (cultivated from Nmairieyh, South Lebanon of 400 m altitude) with different concentration of CDDP showed a higher antiproliferative effect on A549 cancer cells than pomegranate juice, cultivated form Baalbek, Lebanon (1170 m of altitude), combined with low−doses of CDDP and taxotere reported in our previous study [13]. The qualitative phytochemical screening tests showed the presence of different bioactive compounds—including alkaloids, terpenoids, and phenols—that might be responsible for the anticancer and antioxidant activities of pomegranate. Those results are generally in agreement with more detailed studies concerning the composition of pomegranate (Table 4) [25].

DPPH antioxidant and phenol content testing showed the antioxidant activity of pomegranate juice. The antioxidant activity of DPPH was more efficient with methanol than with ethanol. This finding suggests that methanol has the strongest antioxidant activity in pomegranate extract [13]. The total phenol content of pomegranate juice extract was found to be elevated. This was slightly higher than the results previously reported by Ricci et al. [26]. A difference that may be due to cultivar or growth conditions [27].

Caravaca et al. reported 151 phenolics, 65 anthocyanin, anthocyanin–flavanol, and flavanol–anthocyanin adducts in pomegranate juice from Spain [28].

The treatment of A549 cells for 24 h with lyophilized pomegranate juice in a dose dependent manner decreased their viability which reflects the anticarcinogenic activity of pomegranate.

Pabla and Dong showed that treatment with CDDP alone was not completely harmless [23]. Hence, minimizing chemotherapeutic drug dosages and combining it with pomegranate might attenuate their side effects while maintaining the same antineoplastic activity. In this study, when pomegranate (150 µg/mL) was combined with low dose of CDDP (8 µg/mL), cell viability decreased by 33%. While, cell viability decreased by 64% when pomegranate (300 µg/mL) was combined to the same dose of CDDP when compared to CDDP or pomegranate juice alone. Thus, pomegranate enhanced the antineoplastic effect of low dose of CDDP. In line with these results, Yu et al. showed that flavonoid significantly enhanced the chemosensitivity of CDDP in vivo and in vitro. They found that A549/CDDP (resistant to CDDP) cells not only acquired epithelial–mesenchymal transition (EMT) phenotype, but also showed increased NF−kB activity compared with A549 cells (sensitive to CDDP).

Zhao et al. showed that these compounds increase the production of IL−21 expressed in CD8, NK cells, and B cells which induce division and proliferation of these cells [29]. In addition, pomegranate extract increased the ratio of the CD4+:CD8+ T cell subpopulations in peripheral blood mononuclear cells and inhibited apoptosis of PBMC [30].

Turrini et al. [10] studied the mechanism of action of pomegranate used in the treatment of different types of cancer. It has an antioxidant activity by decreasing reactive oxygen species and increasing glutathione S−transferase. It induces apoptosis by increasing the level of caspace−3, −8, and −9 and decreasing the level of survivin. Pomegranate also plays an important role in inhibiting angiogenesis and metastasis by decreasing the level of the vascular endothelial growth factor (VEGF) [10].

The current results corroborated with the hypothesis that pomegranate, not only enhance antineoplastic effect of CDDP, but may also preserve normal PBMC, in a dose dependent manner. These results might be secondary to the presence of polyphenols in pomegranate juice that may induce the PBMC proliferation.

## 5. Conclusions

This study showed that pomegranate juice contains active compounds such as polyphenols, which induce significant anti−cancer activities against A549 cells. In addition, pomegranate juice may enhance the antineoplastic activity of low dose CDDP on A549 lung adenocarcinoma cells. In addition, we have highlighted that pomegranate juice might preserve normal PBMC from the cytotoxic effect of cisplatin; thus, adding pomegranate to cisplatin may increase the antineoplastic effect of CDDP and attenuate blood toxicity. Nevertheless, in vivo researches are needed to confirm these results.

## Figures and Tables

**Table 1:** Detection of primary and secondary metabolites by phytochemical screening

Plant Constituents	Reagents Added	Color
Phenolic acids	FeCl_3_ (1%) + K_3_ (Fe(CN)_6_) (1%)	Greenish Blue color
Terpenoids	Chloroform + concentrated sulfuric acid	Reddish brown color in the surface
Flavonoids	KOH (potassium hydroxide 50%)	Yellow
Quinones	HCl concentrated	Precipitate or yellow color
Alkaloids	Dragendroff reagent	Reddish Orange precipitate/turbidity
Tannins	(FeCl_2_ (1%))	Blue color
Resins	Acetone + water + agitation	Turbidity
Saponins	Vigorous shaking	Layer of foam
Reducing sugar	Water + fehlings (A+B) + boil	Brick-red precipitate
Anthraquinones	HCl (10%) + boil	Precipitate
Proteins and amino acids	Ninhydrin (0.25%) + boil	Blue color
Phlabotannins	HCl (1%) + boil 5 min + cooling	Red precipitate
Flavanones	H_2_SO_4_ concentrated	Purple red color
Diterpenes	Copper sulfate	Green color
Sterols and steroids	Chloroform + H_2_SO_4_ concentrated	Red color of upper layer + greenish yellow fluorescence in acidic layer
Anthocyanins	NaOH (10%)	Blue color
Lignines	Safranin	Pink color
Cardiac glycosides	Acetic acid glacial + FeCl_3_ (5%) + H_2_SO_4_ concentrated	Purple ring + brown ring + green ring
Fixed oils and fatty acids	Spot test	Oil spot

**Table 2:** Chemical composition of pomegranate juice.

Bioactive Molecule	Result
Reducing sugar	+
Anthraquinones	−
Proteins and amino acids	−
Phlabotannins	−
Alkaloids	+
Tanins	+
Resins	−
Terpenoids	+
Flavonoids	+
Quinones	−
Sterols et steroids	+
Diterpenes	−
Anthocyanins	+
Flavanones	+
Lignins	+
Cardiac glycosides	−
Saponins	−
Phenols	+
Fixed oils and fatty acids	−

+: presence, −: absence.

**Table 3:** Carbohydrates, phenol, and dry matter content in pomegranate juice

Test	Content
Amount of carbohydrates	69.29%
Total phenol content	6.93 mg GAE/g
The percentage of dry matter	81.1463%

**Table 4:** Different classes of phytochemicals identified from pomegranate juice.

Classes	Phytochemicals
Ellagitannins, gallotannins, and derivatives	Brevifolin, Brevifolin carboxylic acid, Brevifolin carboxylic acid 10−monopotassium sulphate, Castalagin, Casuariin, Casuarinin, Corilagin, Isocorilagin, Hippomanin A, Gemin D
Flavonoids	Rutin (Quercetin−3−O−rutinoside), Quercetin−3,40−dimethyl ether 7−O−_−L−arabinofuranosyl(1−6)−_−D−glucoside, Cyanidin, Chrysanthemin (Cyanidin−3−O−glucoside), Cyanin (Cyanidin−3,5−di−O−glucoside), (Procyanidin A2, Procyanidin B1, Procyanidin B2, Procyanidin B3
Lignans	Conidendrin, Isohydroxymatairesinol, Isolariciresinol, Matairesinol
Triterpenoids and phytosterols	Punicanolic acid, Ursolic acid, Campesterol, Cholesterol
Fatty acids and lipids	Caproic acid (Hexanoic acid), Caprylic acid (Octanoic acid), Capric acid
Akaloids and indolamines	*N*−(2′,5′−Dihydroxyphenyl)pyridinium chloride, Hygrine, Norhygrine, Pelletierine, *N*−Methylpelletierine, Norpseudopelletierine, Pseudopelletierine, 2−(2′−Hydroxypropyl)−∆^1^piperideine, 2−(2′−Propenyl)−∆^1^piperideine, Punigratane (2,5−Diheptyl−*N*−methylpyrrolidine), Sedridine, Melatonin, Serotonin, Tryptamine
Organic acids and phenolic acids	Ascorbic acid, Citric acid, Fumaric acid, L−Malic acid, Oxalic acid, Quinic acid
